# Use of Abandoned Copper Tailings as a Precursor to the Synthesis of Fly-Ash-Based Alkali Activated Materials

**DOI:** 10.3390/ma18173926

**Published:** 2025-08-22

**Authors:** Arturo Reyes-Román, Tatiana Samarina, Daniza Castillo-Godoy, Esther Takaluoma, Giuseppe Campo, Gerardo Araya-Letelier, Yimmy Fernando Silva

**Affiliations:** 1 Department of Mining Engineering, University of Antofagasta, Avenida Angamos 601, Antofagasta 1270300, Chile; daniza.castillo@uantof.cl; 2 School of Technology, Kajaani University of Applied Sciences, Ketunpolku 1, 87101 Kajaani, Finland; tatiana.samarina@kamk.fi; 3 Outokumpu Stainless Oy, Terästie, 95490 Tornio, Finland; esther.takaluoma@outokumpu.com; 4 DIATI—Department of Environment, Land and Infrastructure Engineering, Politecnico di Torino, 10129 Torino, Italy; giuseppe.campo@polito.it; 5 School of Civil Construction, Faculty of Engineering & Concrete Innovation Hub UC (CIHUC), Pontificia Universidad Católica de Chile, Santiago 7820436, Chile; gerardo.araya@uc.cl (G.A.-L.); yimmy.silva@uc.cl (Y.F.S.)

**Keywords:** copper tailings, alkali-activated materials, secondary raw materials

## Abstract

This study evaluated the feasibility of reusing abandoned copper mine tailings (Cu tailings) as a precursor in the production of fly-ash-based alkali-activated materials (FA-AAMs). Two formulations were developed by combining FA and Cu tailings with a mixture of sodium silicate and sodium hydroxide as alkaline activators at room temperature (20 °C). Formulation G1 consisted of 70% Cu tailings and 30% fly ash (FA), whereas G2 included the same composition with an additional 15% ordinary Portland cement (OPC). The materials were characterized using X-ray fluorescence (XRF), -X-ray diffraction (XRD), field emission scanning electron microscopy with energy-dispersive spectroscopy (FESEM-EDS), and particle size analysis. While FA exhibited a high amorphous content (64.4%), Cu tailings were largely crystalline and acted as inert fillers. After 120 days of curing, average compressive strength reached 24 MPa for G1 and 41 MPa for G2, with the latter showing improved performance due to synergistic effects of geopolymerization and OPC hydration. Porosity measurements revealed a denser microstructure in G2 (35%) compared to G1 (52%). Leaching tests confirmed the immobilization of hazardous elements, with arsenic concentrations decreasing over time and remaining below regulatory limits. Despite extended setting times (24 h for G1 and 18 h for G2) and the appearance of surface efflorescence, both systems demonstrated good chemical stability and long-term performance. The results support the use of Cu tailings in FA-AAMs as a sustainable strategy for waste valorization, enabling their application in non-structural and moderate-load-bearing construction components or waste encapsulation units. This approach contributes to circular economy goals while reducing the environmental footprint associated with traditional cementitious systems.

## 1. Introduction

In Chile, the production of copper (Cu) concentrates is primarily conducted using froth flotation, which produces tailings as by-products [1]. According to the Chilean Geological Survey [2], approximately 537 million tons of Cu tailings are deposited annually in Chile, and this amount is expected to continue increasing. For instance, in 2023, a total of 2,131 tailings deposits were reported, of which 160 were abandoned. A previous study conducted in the northern Chile has determined that abandoned tailings are causing soil contamination with hazardous elements [3,4]. Potentially toxic elements (PTEs) typically found in Cu tailings—such as Cu, Pb, Zn, and As—raise significant environmental concerns due to their high toxicity, persistence, and ca bioaccumulate potential, which may threaten both human health and ecosystems. When tailings are not properly managed, they can contaminate soil and groundwater through leaching processes, particularly under acidic or oxidizing conditions [5]. Prolonged exposure to these PTEs has been associated with adverse neurological, renal, and carcinogenic effects in humans, as well as reduced biodiversity in both aquatic and terrestrial environments [6]. In regions where mine tailings remain abandoned or insufficiently controlled, the associated long-term environmental risks can be considerable. This study highlights the potential of alkali-activated materials as a dual-purpose solution, facilitating the reuse of industrial residues and enabling the immobilization of PTEs within chemically stable matrices. This strategy is consistent with sustainable waste management practices and supports the development of environmentally responsible, low-carbon construction materials with remediation potential [7,8].

The chemical and mineralogical composition of mine tailings—regardless of whether they originate from copper, gold, or other ore types—is highly variable and influenced by the original ore mineralogy, the processing techniques employed, and local geochemical conditions [9,10]. For example, tailings derived from sulfide-rich Cu ores tend to be enriched in silica, iron (Fe) oxides, and elements such as As, while those from oxide or laterite-type deposits may contain higher proportions of alumina or calcium-bearing minerals [11,12]. These differences govern not only the reactivity and stabilization behavior of the tailings, but also determine their suitability for reuse in applications such as alkali-activated materials and geopolymers [13]. The Cu tailings generated in Chile exhibit both compositional and environmental attributes that differentiate them from those produced in other mining countries. These tailings, largely derived from flotation of sulfide ores such as chalcopyrite and enargite, are characterized by a high silica content (typically > 60 wt%) and low levels of alumina and calcium oxides, which renders them poorly reactive in alkaline media without prior treatment [9,14]. In contrast, Cu tailings from regions such as China and Australia may contain higher levels of reactive aluminosilicates or calcium-bearing phases, enhancing their alkali activation potential [10,11]. A distinctive feature of Chilean tailings is their elevated As content, often exceeding 1000 mg/kg, due to the natural abundance of As-bearing minerals like enargite [3,15,16]. This creates serious environmental challenges and necessitates the use of stabilization strategies such as geopolymerization. Moreover, the hyper arid climate of northern Chile limits mineral hydration and weathering, preserving a largely crystalline mineral structure dominated by quartz, which further reduces their chemical reactivity [13]. In comparison, tailings from other regions often exhibit a higher amorphous phase content, making them more suitable for direct alkali activation without extensive pretreatment [12,17].

The use of tailings as a secondary raw material has been reported in a variety of applications, including the synthesis of supplementary cementitious materials (SCM) [9,18,19] where tailings partially replace cement [20,21]; preparation of backfilling materials for mining operations [22,23]; additive aggregates in concrete [10,24,25]; and the manufacture of alkali-activated materials (AAMs) and geopolymers, including hybrid alkali activated cements (HAACs) [26]. The use of tailings in some of the applications described above could help reduce carbon emissions by minimizing the demand for cement and natural aggregates [27,28,29]. Additionally, the use of tailings as precursor or aggregates in the synthesis of AAMs could help address the problem of tailings disposal caused by the increasing amounts of waste generated by the mining industry, as well as the issue of abandoned mine tailings [30,31]. AAMs are characterized by high mechanical strength, good durability, and resistance to acidic media and thermal impacts. They are also capable of encapsulating potentially toxic elements (PTEs) within their structure [31], which is essential for stabilizing mine tailings. It is known that depending on whether the calcium content is low or high, aluminosilicate gels will form, which are known as sodium aluminosilicate hydrate (N-A-S-H) or calcium (alkali) aluminosilicate hydrate (C-(N)-A-S-H) structures, respectively [32]. AAMs with low calcium content are referred to as geopolymers, which are obtained from amorphous or crystalline aluminosilicate materials [28,33] activated by alkaline agents. The most commonly used alkaline activators are sodium or potassium hydroxides and silicate solutions [34]. The molar ratios of Si/A, Si/Na, and Na/Al, along with the liquid-to-solid ratio, are the main factors that influence the mechanical strength, immobilization capacity, and durability of geopolymers [34,35,36]. The silica and aluminum content of tailings are poorly soluble in alkaline media due to their crystalline structure [11,31,37,38], and their degree of dissolution varies significantly depending on the type of minerals they contain [10,39]. Since mining tailings are largely unreactive [10,40], their use in the synthesis of AAMs would require pretreatment methods, such as mechanical, thermal, and thermochemical activation [41,42,43], which increase the specific surface area and transform the chemical species in the tailings into amorphous and soluble phases [44]. There are several studies where gold tailings [45], iron tailings [46], and coal mining waste [47] were used as aggregates to combine with volcanic glass, metakaolin, blast furnace slag, or fly ash (FA) [48]. It was observed that the addition of tailings either did not affect or enhance the compressive strength and density of the matrix, in addition to decreasing the setting time. Geopolymers made with mine tailings and FA [11] exhibited denser microstructures when up to 30% sodium silicate was used [49] or if higher concentrations of NaOH were used [17,18,19,20,21,22,23,24,25,26,27,28,29,30,31,32,33,34,35,36,37,38,39,40,41,42,43,44,45,46,47,48,49,50,51]. The case of geopolymers made with Au and Cu tailings mixed with aluminum residues was reported, where high compressive strength was explained by having reached optimal Si/Al and Na/Al ratio [13,52]. There is still a lack of research on the feasibility of using abandoned Cu mine tailings with inherently low calcium and aluminum content as precursor components in FA-AAMs. Most previous studies on mine tailings in alkali-activated systems have focused on enhancing reactivity by incorporating reactive binders such as slag or FA, or by applying mechanical, thermal, or chemical activation methods [38]. Recent studies have shifted attention toward more sustainable alternatives that enable the direct use of unprocessed or minimally treated tailings in alkali-activated matrices [53]. To the best of the authors’ knowledge, this is the first study addressing that gap by evaluating the performance of untreated Cu tailings, collected from an abandoned site in northern Chile, when used as inert fillers in FA-AAM formulations. The objective of this study was to determine the potential of an abandoned Cu mine tailing to be used as an aggregate in the preparation of a FA-AAMs. To achieve the abovementioned objective, this study is organized as follows: Section 2 presents the physical, and chemical properties of raw materials as well as the preparation, physical, mechanical and leaching properties of FA-AAMs. Section 3 details the results of the experimental procedures, along with a detailed discussion of their implications for materials applications. Finally, Section 4 presents the main conclusions of this study as well as recommendations for future studies.

## 2. Materials and Methods

### 2.1. Sampling of Raw Materials

**Figure 1:**
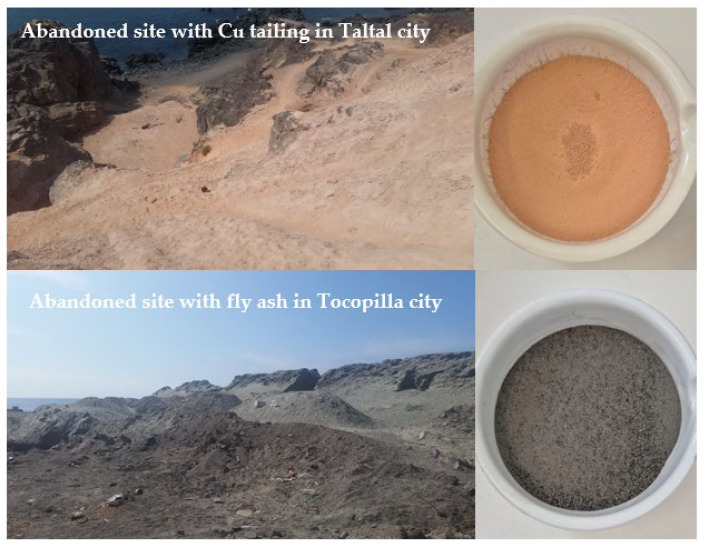
Images of the abandoned locations from which Cu tailings (**top**) and FA (**bottom**) samples were obtained.

Cu mine tailings samples were obtained from an abandoned site (UTM Datum WGS 84. 7190181 N; 351516 E) located in the Taltal commune, Antofagasta Region of Chile, where informal processing of copper ore took place more than 40 years ago and where the residues remain untreated to this day [3,15]. The FA generated by a former coal-fired power plant was collected from an abandoned site in the city of Tocopilla (Figure 1). A general, ordinary Portland cement (OPC) classified as Type I according to ASTM C150 [54], was supplied by Cementos Bío Bío (Talcahuano, Chile) and was employed in the formulations. The sampling of the abandoned Cu mine tailings and fly ash was carried out following established environmental management protocols and waste characterization procedures [55,56]. Organic compounds, similar in nature to the components of residual flotation reagents commonly found in tailings, may influence cement hydration through pH changes, surface modification, and interference with hydration products [57]. However, no specific information was available for the Cu tailings used, as they were discarded decades ago. Therefore, the study focused on evaluating their applicability in alkali-activated systems rather than analyzing residual flotation chemicals.

### 2.2. Raw Materials and FA-AAMs Characterization

The samples of Cu mine tailing, FA, OPC, and FA-AAMs were characterized using both chemical and physical methods. The pH was measured after adding KCl (1 M, 12.5 mL) to 5 g of dried sample, following a previously established protocol [9]. After shaking for 1 h, the sample pH was measured by a pH-meter (WTW multimeter (Profline pH 3110 set 2) equipped with a SenTix 41 pH electrode (WTW, Weilheim, Germany). The chemical composition was determined using both by X-ray fluorescence (XRF), performed on an S4 Explorer Bruker X-Ray Spectrometer (Bruker, Billerica, MA, USA), and inductively coupled plasma optical emission spectrometry (ICP-OES), conducted with an Agilent Model 5110 SVDV spectrometer (Mulgrave, Australia), following aqua regia extraction. The mineralogical composition was determined by X-ray diffraction (XRD) using a Bruker D8 Advance diffractometer equipped with a Cu Kα radiation source (λ = 1.5406 Å) and a LynxEye linear detector. Prior to analysis, powdered samples were finely ground manually using an agate mortar to obtain a particle size below 75 µm. Scanning was carried out over a 2θ range of 5° to 70°, with a step size of 0.02° and a counting time of 1 s per step. The acquisition parameters were optimized to ensure high-quality signal acquisition and resolution. Phase identification and quantification, including estimation of the amorphous content, were performed using the Rietveld refinement method with TOPAS V4.2 software. The analysis was carried out by refining a model against the experimental diffractogram, integrating crystallographic information of the identified phases and the instrument’s physical configuration parameters. The weight percentage (wt.%) of each species was calculated based on the refined relative peak intensities and considering the crystallographic density of each mineral phase. The diffractogram interpretation was supported by reference to the ICDD (International Centre for Diffraction Data) database, employing PDF reference cards to assist in phase identification. The ICDD card numbers associated with each detected phase are listed to ensure data traceability and enable direct comparison with standard crystallographic references. Field Emission Scanning Electron Microscopy (FESEM) with Energy Dispersive Spectroscopy (EDS) (FEI Quanta 250 FEG-SEM, Thermo Fisher Scientific, Hillsboro, OR, USA) was used to study the microstructure, surface morphology, and elemental composition at selected points. The samples were prepared by mounting them on double-sided conductive carbon tape and coating them with approximately 10 nm of gold via sputtering to minimize surface charging. Given that the analyzed structures exceeded 10 µm in thickness, any contribution from the carbon tape substrate is considered negligible. Moreover, analyses were performed at an accelerating voltage of 20 kV, further reducing the influence of the thin gold layer, although its presence is still discernible in the spectra. Particle size distribution (PDS) analysis was analyzed by LSD using a Malvern Mastersizer 2000 instrument (Malvern Panalytical, Worcestershire, UK). Organic matter was determined by loss-on-Ignition (LOI). The LOI corresponds to the percentage weight loss of a sample (2.5 g) dried in a crucible heated to 550 °C for 1 h in a muffle furnace. The Chilean regulation related to cement, NCh 148.Of21 [58], which specifies the chemical and physical criteria required for various types of cement, was used as a reference to analyze the chemical composition of both the Cu tailings and the FA.

### 2.3. Preparation of FA-AAMs

Two formulations, G1 and G2, were prepared using Cu mine tailings and FA as raw materials. G1 excluded OPC, whereas G2 incorporated 15% OPC in its composition. The incorporation of OPC aimed to avoid the need for thermal curing, enabling ambient-temperature setting and hardening, while enhancing both fresh and hardened-state properties. Thermal and mechanical activation methods—such as calcination and grinding—were intentionally excluded from the treatment of Cu tailings in this study to minimize the environmental footprint of the final material. High-energy treatments are recognized for improving reactivity; however, they also significantly increase energy consumption and carbon emissions, which can undermine the environmental advantages associated with the use of industrial by-products [28,38]. Instead, their low reactivity was compensated by blending with reactive FA, which effectively supported the geopolymerization process [10,11]. It is worth noting that an initial trial using 100% Cu tailings was conducted, but the paste was neither workable nor thixotropic enough, which has been previously reported [59]. A ratio of 70% Cu tailings to 30% FA was selected based on preliminary tests, which indicated that tailing-only mixtures exhibited higher water demand and insufficient reactivity. This composition was found to produce a workable paste. In this case the incorporation of FA as an additional aluminum source is recommended to achieve a balanced network structure [60]. The mix proportion of each formulation are presented in Table 1. The activating solution was prepared by combining sodium silicate (SS) (1.58 g/cc) with sodium hydroxide (SH) pellets to achieve a SS modulus of 1.5. The activating solution was used only after reaching room temperature (approximately 20 °C). A silicate modulus of approximately 1.5 is frequently employed in geopolymer formulations, as it provides an optimal balance between precursor dissolution and gel network development, resulting in a stable, cross-linked aluminosilicate matrix [61]. Geopolymers synthesized at this ratio have demonstrated enhanced compressive strength and microstructural density [62], along with improved durability and resistance to environmental degradation [63]. Furthermore, this composition supports adequate workability and accelerated setting times, which are advantageous for practical implementation [64].

The Cu tailings and FA were blended in a vertical mixer (IKA, Staufen im Breisgau, Germany, model Eurostar 100) for 2 min, after which the activating solution was incorporated. The amount of water added was adjusted to achieve optimal paste workability. The Si/Al, and Na/Al molar ratio were calculated for each paste. The 50 × 50 × 50 mm specimens were prepared in accordance with ASTM C109/C109M [65], which recommends 2-inch (50 mm) cubes for compressive strength testing of hydraulic cement mortars. The paste was thoroughly mixed until the desired consistency was achieved, then cast into these molds. This smaller size enabled efficient material use and easier handling, while still providing reliable compressive strength data when standard procedures were followed. The molds were vibrated for 5 min to remove any trapped air bubbles and then covered with plastic film to prevent water evaporation. After 24 h, the samples were demolded and cured in a humid environment (>80% RH) at room temperature, without thermal treatment, until the time of testing. Each formulation was prepared in triplicate.

### 2.4. Physical and Mechanical Properties of FA-AAMs

The determination of consistency, setting time, absorption, porosity, and compressive strength of FA-AAMs was conducted according to the current applicable ASTM and Chilean standards. Consistency was determined following ASTM C187-23 [66], while setting time was measured using a Vicat apparatus according to ASTM C191-19 [67]. To determine absorption and porosity, a paste sample was first oven-dried until it reached a constant weight, which was recorded as the dry weight. The sample was then submerged in water for a specified period (typically 24 h), after which it was weighed again to determine the saturated weight. The water absorption percentage was calculated using the formula showed in Equation (1):

(1) $$Absorption\left(\%\right)=\frac{\left(Saturated\;weight-Dry\;weight\right)}{Dry\;weight}\times 100$$

Although there is no specific Chilean standard for directly measuring mortar porosity, it was inferred from water absorption and bulk density tests. Following ASTM C20-00 [68], the procedure required weighing the specimen in three conditions: dry, saturated, and submerged. For the submerged measurement, the sample was placed in a holder that prevented contact with the container walls or base, allowing the accurate measurement of its apparent weight under water, influenced by buoyancy. The porosity calculation is based on Archimedes’ principle and is commonly used to determine open porosity in materials like ceramics, geopolymers, and cementitious composites [69]. The apparent porosity was then estimated using the formula shown in Equation (2):

(2) $$Porosity\left(\%\right)=\frac{\left(Saturated\;weight-Dry\;weight\right)}{\left(Saturated\;weight-Submerged\;weight\right)}\times 100$$

The compressive strength tests were conducted in accordance with ASTM C109/C109M-02 [65]. After curing for 7, 14, 28, 60, 90, and 120 days, specimens of each sample were tested to determine their compressive strengths. The specimens were surface-polished at the ends to ensure they were perfectly flat and parallel prior to testing. The compressive strength was calculated by dividing the maximum load at failure by the cross-sectional area of the specimen. The tests were carried out using a Controls compression machine (Liscate, Milan, Italy) with a 600 kN capacity and a constant loading rate of 1 mm/min. To ensure repeatability and accuracy, the results presented are the average of replicate specimens. The standard deviation from at least three samples was used to quantify the variability in the measurement.

### 2.5. Leaching Test Conducted on FA-AAMs

Leaching tests were conducted in accordance with Chilean Supreme Decree No. 148 [70], which establishes the criteria for classifying solid waste as hazardous and is based on the Toxicity Characteristic Leaching Procedure (TCLP), as described in EPA Method 1311 [71]. The samples were dried at 105 °C, ground to a particle size of less than 9.5 mm, and their pH was measured to determine the appropriate extraction solution. Two extraction solutions are defined: solution #1 (pH ~ 4.93) is prepared with glacial acetic acid and sodium hydroxide, while solution #2 (pH ~ 2.88) is prepared by diluting 5.7 mL of glacial acetic acid directly to 1 L with deionized water. Since the sample’s pH remained above 5.0 after the buffer capacity test, solution #2 was selected. The leaching was performed at a 20:1 liquid-to-solid ratio, rotating at ~30 rpm for 18 ± 2 h. The mixture was then filtered through a 0.45 µm glass fiber filter, and the leachate was acidified for metal analysis using atomic absorption spectroscopy (AAS). The results were compared with regulatory limits to evaluate potential environmental hazards. The purpose of the leaching test was to evaluate the chemical stability of the compounds present in FA-AAMs by assessing their potential to release PTEs.

### 2.6. Statistical Methods

For each experimental test, a minimum of three specimens per formulation were prepared and analyzed to ensure data consistency and reproducibility. The results for consistency, setting time, absorption, porosity, and compressive strength of FA-AAMs are presented as average values derived from these triplicates, with standard deviation values included to quantify the result variability. Although no advanced statistical modeling was applied in this study, the use of replicate measurements enhances the reliability of the findings.

## 3. Results and Discussion

### 3.1. Chemical, Physical and Mineralogical Composition of Raw Materials

**Figure 2:**
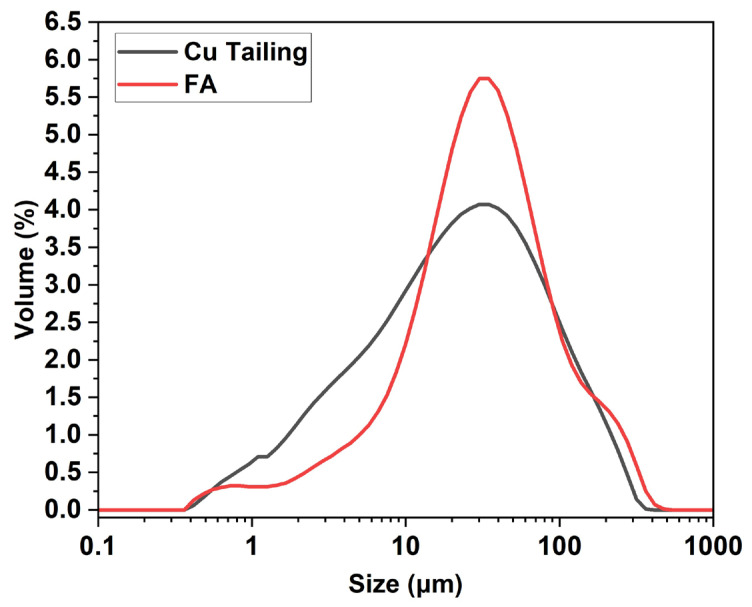
Size particle distribution analysis of raw materials.

**Figure 3:**
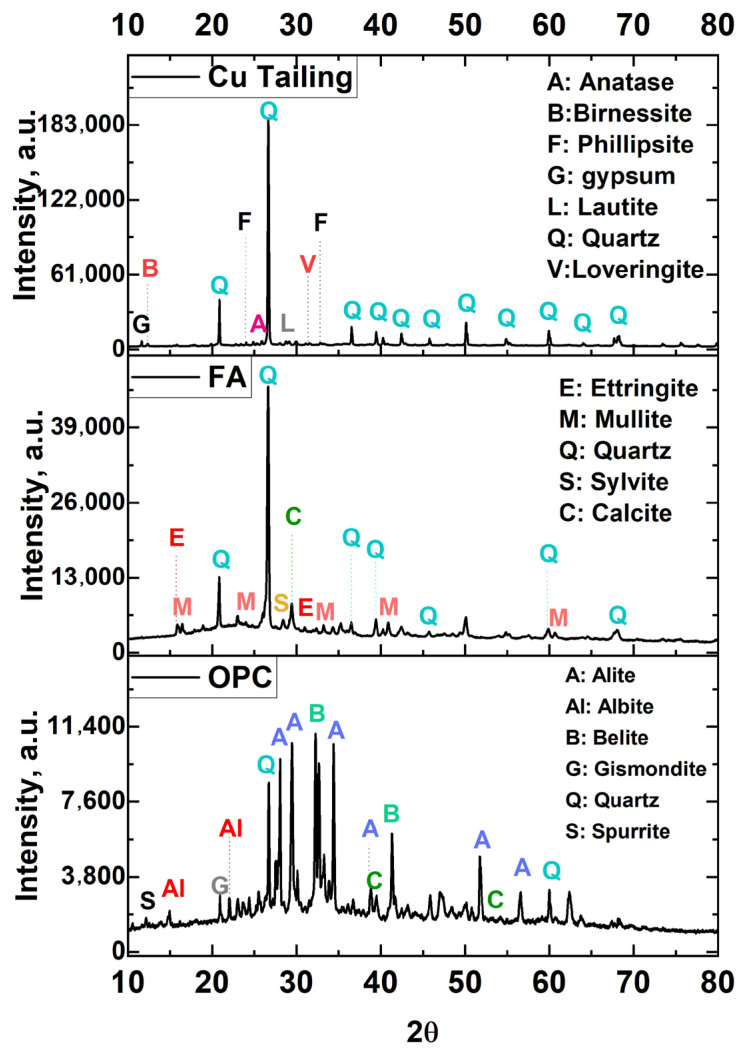
XRD patterns of OPC (**top**), Cu mine tailings (**middle**), and FA (**bottom**) samples.

Table 2 summarizes the chemical composition, physical properties, and mineralogical characteristics of the raw materials used in the synthesis of FA-AAMs, namely Cu tailing, FA, and OPC. The Cu tailings primarily consists of silicon and aluminum oxides, followed by iron, barium, calcium, and potassium oxides. The FA exhibited a high quartz content, along with notable amounts of alumina, calcium oxide sulfur oxide, iron oxide, and potassium oxide. In the case of Cu tailings, the Al_2_O_3_ content (5.35%) was lower, while the SiO_2_ content (77.82%) was higher compared to FA, which contained 13.12% Al_2_O_3_ and 55.39% SiO_2_. According to the ASTM C618-19 [72], the FA is considered a low-calcium material because it contains less than 10% calcium oxide (CaO). However, it still had a higher calcium content (9.73%) than the Cu tailings (0.86%). Due to the low calcium content of these materials the resulting formulations were expected to form geopolymers with low crystallinity or predominantly amorphous structures [73,74]. The measured pH values of Cu tailing and FA were 7.5 and 10.7, respectively. This characteristic is due to the presence of oxides such as CaO and magnesium oxide (MgO), which increase the pH levels of these materials. The pH of tailings depends on the oxidation process of acid-forming sulfides and acid neutralization by the dissolution of minerals, particularly carbonates [75]. The LOI value indicates the amount of volatile substances, including unburned materials and calcite, that are released during the firing process [59]. The measured organic matter contents of the Cu tailings and FA samples were 5.7 wt.% and 11.8 wt.%, respectively. It is known that organic matter contents as low as 1% can delay the setting of soil–cement mixtures by disrupting with cement hydration [76]. The specific gravity of the Cu tailing particles and FA was 2.4 and 1.9, respectively, indicating that the Cu tailing particles are denser than the FA. The Cu tailings analyzed in this work exhibited a lower specific gravity (2.49) compared to traditional fine aggregates such as natural sand, which generally ranges between 2.60 and 2.70 [77]. The particle size distribution indicated that the Cu tailings were finer than FA, with 90% of the particles measuring less than 105.2 µm, whereas for FA, 90% of the particles were below 111.2 µm. Furthermore, Cu tailings demonstrated a greater specific surface area (0.809 m^2^/g) than FA (0.534 m^2^/g), confirming the slightly coarser nature of the latter (Figure 2). Regarding direct reactivity testing of the Cu tailings, no dedicated assessments—such as alkaline dissolution experiments, or solubility tests—were performed. However, their low reactivity was inferred from their predominantly crystalline structure and minimal amorphous content, as reveled by XRD analyses. Future research should incorporate standardized reactivity evaluations, such as alkaline solubility and pozzolanic activity tests, to more accurately assess the reactive potential of these materials. The MgO and SO_3_ contents in the Cu tailings and FA were within the maximum allowable limit of 5.0%, as established by the Chilean standard [58] for hydraulic cements. This compliance confirms the potential suitability of these materials as supplementary cementitious components. Maintaining these oxide concentrations below the specified thresholds is essential to avoid undesirable volumetric expansion phenomena, such as that caused by the formation of expansive phases [78]. The concentration of Na_2_O in the Cu tailings and FA was below the 5% limit typically recommended for these materials to avoid alkali–silica reactions [9]. Although in this work the Cu tailings were primarily used as a precursor material for geopolymer synthesis, it is acknowledged that some particles fall within the typical size range of fine aggregates. This heterogeneous granulometry could influence setting behavior and physical–mechanical properties, potentially affecting reactivity, packing density, and strength development. The finer granulometry and increased surface area of the tailings are advantageous for enhancing early mechanical performance, as they promote more efficient particle packing and accelerate the geopolymerization or hydration reactions, thus leading to a more compact matrix [9,77]. These characteristics support the application of both materials in construction, given the positive influence of their fineness on early strength development [77,79]. The diffractogram of the Cu tailings exhibited more pronounced peak intensities than that of the FA, indicating a higher crystallinity in the tailings. In contrast, the lower intensity peaks observed for FA reflect its more amorphous nature (Figure 3).

The mineralogical composition of the Cu tailing, FA, and OPC samples is shown in Table 2. The quantitative analysis enabled us to determine the relative amounts of both crystalline and amorphous phases present in each sample. The XRD patterns of the Cu tailings revealed the presence of crystalline phases such as quartz (64.56%), along with various minor mineral species including phillipsite-K (7.77%), loveringite (5.05%), periclase (4.69%), lautite (3.39%), natisite (2.67%), sturmanite (2.64%), greigite (2.58%), grossite (1.73%), virgilite (1.33%), synthetic birnessite (1.26%), and gypsum (0.87%). These mineral phases reflect the extent of weathering undergone by the Cu tailings, which have remained exposed for decades at a coastal site in northern Chile [3,15]. For example, the presence of phillipsite-K (7.77%), a zeolitic mineral, indicates that the tailings may have been subjected to significant environmental weathering. In contrast, the FA sample was primarily composed of quartz (24.37%) and mullite (4.18%), the latter being a crystalline phase formed during high-temperature coal combustion. While quartz is a largely inert phase, mullite is a crystalline component that may enhance the structural stability of FA within cementitious systems. The presence of ettringite (2.48%) in the FA indicates the existence of sulfate phases, which may participate in secondary reactions during the hydration process in cementitious systems.

While no amorphous content was detected in the Cu tailings, the FA exhibited 64.4% amorphous material, which is known to be highly reactive in geopolymer systems. The results suggest that Cu tailings are less reactive than FA, supporting the conclusion that they are more suitable for use as inert aggregates [80,81]. In contrast, the mineralogical characteristics of FA—particularly its higher amorphous content—indicate a greater tendency to dissolve in alkali-activated systems Additionally, Chilean tailings tend to have a fine particle size distribution (d90 < 150 µm), which makes them suitable for use as filler aggregates but generally unsuitable as standalone precursors in alkali-activated binder systems, due to their low amorphous content and poor alkali reactivity [82]. Prior research has indicated that low-reactivity mine tailings can serve as passive fillers when used in combination with active binders such as FA [83,84].

The FA used in this study, classified as Class F, contains over 70% of silicon, aluminum, and iron oxides, which confer pozzolanic properties. However, due to its low calcium content, it may require an external calcium source—such as lime or OPC—to develop cementitious behavior. Under alkaline conditions, it can also participate in hydration reactions. The OPC sample contained a significant amount of larnite (19.93%) and gismondine (12.64%), both important phases in cement chemistry. Compounds with similar properties to larnite, such as belite (C_2_S), are known to contribute to strength development by reacting with water during the hydration process to form calcium silicate hydrates (C–S–H), particularly at later curing stages when their reactivity becomes more significant [85]. Gismondine, a hydrated calcium aluminum silicate mineral, may contribute to the development of stable hydration products by acting as a reactive aluminosilicate phase. Additionally, the presence of calcite (13.87%) may serve as a supplementary calcium source, potentially promoting the formation of C–S–H [21]. In this context, the addition of OPC likely served as the external calcium source required to facilitate these processes. The use of low-calcium Cu tailings and FA provided an opportunity to study the behavior of low-Ca alkali-activated systems, where the only additional calcium source was the OPC incorporated into the G2 formulation. However, incorporating high-calcium FA may offer a promising strategy to enhance early-age strength and promote the formation of calcium (alumino) silicate hydrate (C–(A)–S–H) gels, which are known to improve matrix densification and mechanical performance. Previous studies have demonstrated that high-Ca FA can act synergistically with aluminosilicate precursors, enhancing reactivity and refining the microstructure in alkali-activated systems [7]. The partial replacement of low-Ca FA with high-Ca FA could be explored to assess its effects on gel chemistry, early strength development, and long-term durability.

**Figure 4:**
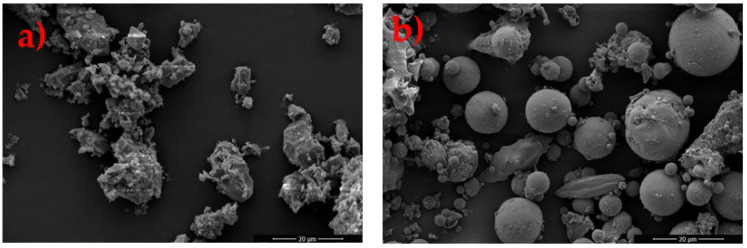
FESEM micrograph of as-received: (**a**) Cu tailing powder and (**b**) FA samples.

Figure 4 presents FESEM micrographs of Cu tailing and FA. The micrograph of Cu tailings (Figure 4a) reveals irregularly shaped particles with a coarse surface texture and extensive voids, features commonly associated with mine tailings. These irregular morphologies likely result from mechanical grinding, crushing, or the natural disintegration of minerals during the mining process. The FA particles (Figure 4b) display predominantly spherical and uniformly sized granules, a characteristic morphology of fly ash, surrounded by smaller particulates. Additionally, the presence of irregularly shaped particles and agglomerates may suggest incomplete combustion or fragmentation of larger particles.

### 3.2. Mineralogical Composition of FA-AAMs

**Figure 5:**
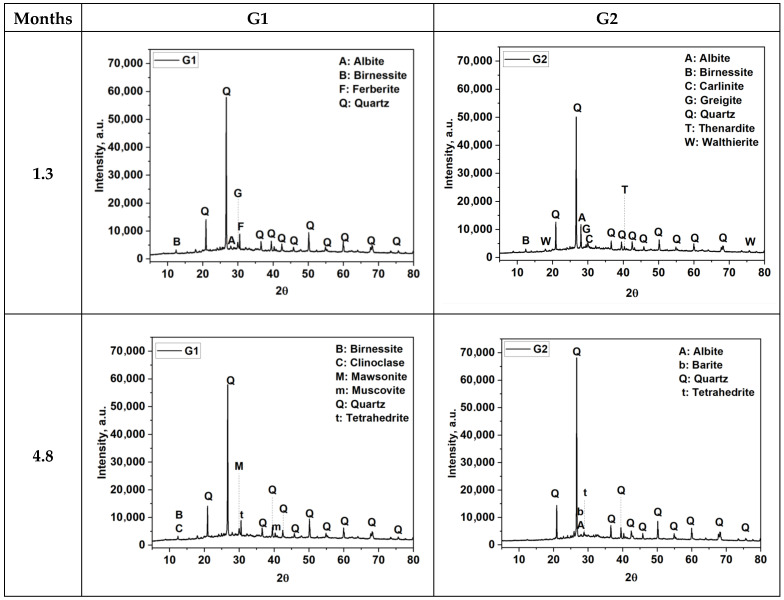

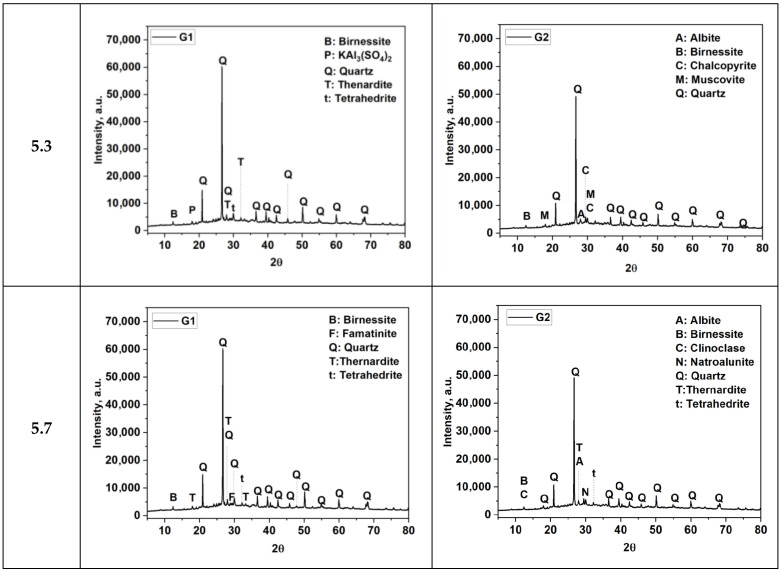
XRD diffractograms of synthesized G1 and G2 samples at various curing times.

The XRD diffractograms presented in Figure 5 indicate that the intensities of the main crystalline peaks—particularly those corresponding to quartz (Q)—remain largely unchanged over the curing periods for both G1 and G2 formulations. In addition, birnessite and several other minor phases are identified. On the other hand, an amorphous halo is evident in all diffractograms between 20° and 40° 2θ, indicating that the products formed during geopolymerization are predominantly in the gel phase. The observed increase in compressive strength over time can be explained by complementary mechanisms that contribute to microstructural development. One possible explanation is the gradual densification of the matrix through slow and progressive geopolymerization processes, particularly involving the amorphous phase, which may not alter the crystalline signature. Additionally, prolonged curing facilitates continued ionic diffusion, pore structure refinement, and a reduction in internal defects, all of which contribute to improved mechanical performance. Thus, strength development may proceed independently of clear mineralogical changes observable by XRD. During the curing periods analyzed, several crystalline phases were identified in both formulations, including quartz (Q), birnessite (B), and tetrahedrite (T), some of which are listed in the table. The persistent detection of these phases across all curing times reflects the stability of the crystalline components in both systems, while also indicating differences in mineral assemblage induced by the presence of calcium-bearing precursors in G2.

Table 3 shows the mineralogical compositions of samples G1 and G2, analyzed as a function of curing time. As observed in the diffractograms, quartz appeared to be one of the dominant phases in both samples, with its percentage increasing gradually over the curing period. The results indicated that after 5.7 months, quartz remained the main crystalline phase in both G1 and G2 throughout curing, along with phases such as muscovite, clinoclase, and thenardite. Additionally, a clear increase in the content of albite, aluminum fluoride, birnessite, and natroalunite was observed in the case of G2. The formulation G2 also contained a higher proportion of amorphous material (41.81 wt.%) compared to G1 (33.25 wt.%), which likely contributed to its enhanced early strength, although changes in amorphous content over time were not quantified. Initially, the G1 and G2 samples exhibited a high presence of amorphous material, though it is more prominent in the G2 sample (41.81%) compared to the G1 sample (33.25%). However, no data on amorphous content were reported for later stages of curing. The amorphous phase plays a crucial role at the early stages, starting at relatively high levels and then gradually transforming into crystalline phases such as quartz and albite as the curing process continues. The initial abundance of amorphous material likely facilitated faster reaction kinetics and phase transformations during the early stages of curing [34]. Additionally, minerals like albite, muscovite, and clinoclase increased in concentration with time in both samples, suggesting ongoing geochemical transformations. However, some phases such as birnessite and rectorite tended to decrease with curing, indicating potential phase transformations or dissolution. In the case of G2, the inclusion of OPC likely promoted the formation of calcium-rich hydration products such as C–S–H and C–A–S–H gels [86]. These phases are typically poorly crystalline or amorphous and may not be clearly identified by XRD analyses, yet they play a crucial role in enhancing mechanical strength [87]. Overall, G2 appeared to undergo more pronounced mineralogical changes over time, possibly due to differences in composition or reaction kinetics. The presence of OPC appears to accelerate the crystallization of albite, while slightly reducing the quartz content, likely due to the interaction of calcium from OPC with the aluminosilicate network. Additionally, several sulfate-based minerals (e.g., anhydrite, thenardite, and walthierite) appear in varying concentrations in both systems, indicating possible sulfate-related processes, such as the dissolution or crystallization of these salts. The presence of OPC, which introduces additional sulfate, could promote higher levels of thenardite crystallization No quantitative data on amorphous content were reported for later curing times. This omission likely reflects limitations of the XRD method and refinement settings, rather than the complete absence of amorphous phases at advanced stages. As the curing process advances, partial crystallization and matrix densification can reduce the intensity of the amorphous halo or lead to overlapping peaks, making it more difficult to resolve non-crystalline signals. Additionally, variations in background correction and fitting procedures may influence the visibility of broad diffuse humps typically associated with geopolymer gels.

**Figure 6:**
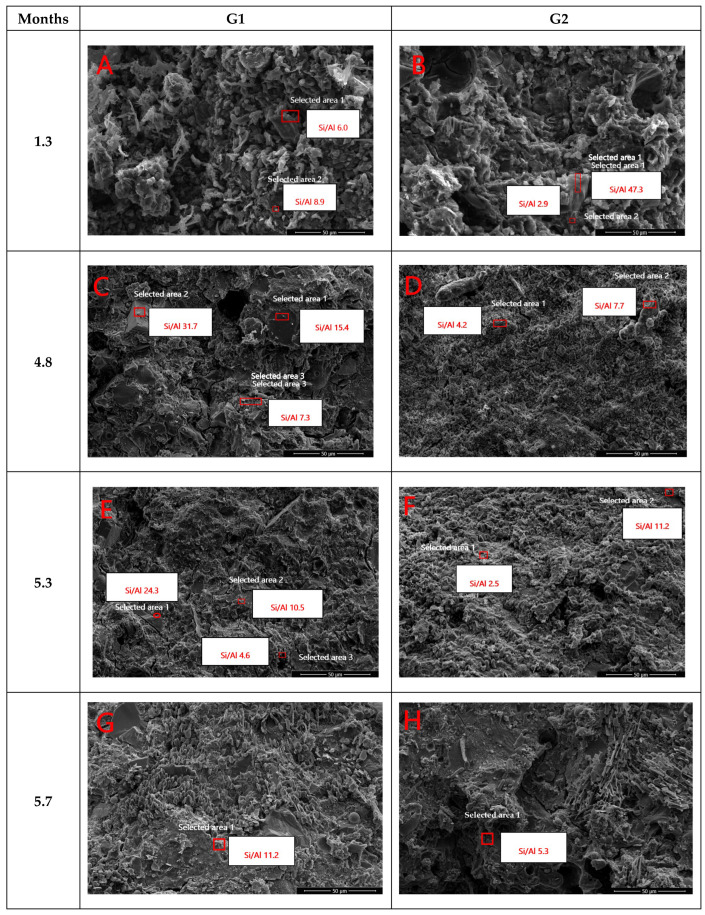
FESEM micrographs of G1 and G2 samples at several curing time. (**A**) G1 at 1.3 months, (**B**) G2 at 1.3 months, (**C**) G1 at 4.8 months, (**D**) G2 at 4.8 months, (**E**) G1 at 5.3 months, (**F**) G2 at 5.3 months, (**G**) G1 at 5.7 months, and (**H**) G2 at 5.7 months. The red square marked in the figure is the location where the EDS analysis was made.

The mineralogical results obtained from XRD analysis were supported by the microstructural observations obtained through FESEM imaging. These mineralogical features support the formation of a more compact and cohesive microstructure in G2, as confirmed by FESEM observations. Albite, as a sodium aluminosilicate, enhances the availability of reactive aluminum and silicon, favoring the development of gels that contribute to matrix densification. The presence of aluminum fluoride suggests greater aluminum stability under alkaline conditions, indirectly supporting gel formation. Birnessite, a manganese oxide with a layered structure, may assist in reducing porosity through secondary reactions and cation immobilization. Natroalunite, which forms under alkaline conditions and contains both aluminum and sodium, indicates a chemical environment conducive to low Si/Al gel formation. Figure 6 shows FESEM micrographs taken for G1 and G2 samples after different curing periods. It is known that the geopolymerization reaction is rapid and occurs within the first few days [19]. However, monitoring the development of the material’s microstructure was considered important to relate these changes to the compressive strength values measured throughout the curing process. In general, G2 exhibited a denser and more uniform microstructure than G1 throughout the curing process, characterized by fewer voids and better particle integration. This enhanced densification could be attributed to the presence of OPC, which promoted additional hydration and gel formation, leading to improved structural performance. After 1.3 months, the micrograph of the G1 sample (Figure 6A) shows a loosely packed surface, with visible voids and particle agglomerates. Some FA spheres and fragments of Cu tailings are distinguishable. In the case of the G2 sample (Figure 6B), the microstructure appears irregular in shapes and surface texture, large unreacted particles, and visible gaps between them. Densification was observed in a Cu tailing-based geopolymer when copper slag was incorporated [88]. The addition of Fe mine tailings to a FA-based geopolymer resulted in densification, which was attributed to the formation of an additional C–S–H phase [89]. The development of compressive strength in FA-based geopolymer concrete is characterized by a significant gain during the initial 24 h, after which the rate of strength increase becomes more gradual [90]. After 4.8 months, the matrix of the G1 sample (Figure 6C) appears more uniform, with fewer visible voids than observed in earlier stages. In contrast, the G2 sample (Figure 6D) shows a more compact and continuous microstructure. After 5.3 months, the G1 sample (Figure 6E) shows increased compaction and a reduction in large, distinct particles, with gel phases expanding and contributing to the densification of the structure. The Cu tailings, still in angular form, become more integrated into the matrix. The G2 sample (Figure 6F) exhibits reduced visible voids and a significantly denser matrix. As the curing continues, the material further densifies, with additional hydration and geopolymerization products forming. After 5.7 months of curing, the G1 sample (Figure 6G) shows a more compact, dense, and smooth matrix, with very few visible voids. In contrast, the G2 sample (Figure 6H) after 5.7 months displays an even more highly compacted, uniform, and well-bonded matrix, with minimal voids and cracks. This suggests that, in both cases, a nearly complete reaction of the components occurred, where the amorphous phases have likely transformed into more crystalline or stable forms.

The calculated Si/Al molar ratios for Cu tailings and FA were 49.4 and 15.3, respectively, both of which markedly exceed the optimal range of 2.5 to 5, typically recommended for geopolymer synthesis [34]. The EDS results presented in Table 4 reveal variations in the Si/Al and Na/Al ratios of G1 and G2 samples over the curing period, indicating progressive chemical and structural development within the geopolymer matrices. The Si/Al ratios tended to be lower in the G2 sample compared to G1, likely due to the higher aluminum content in G2, which could favor the formation of a more balanced and cohesive gel structure. These Si/Al molar ratios were lower than that of the Cu tailings (49.4), but still exceeded the estimated value of approximately 3.31 reported in previous studies [91]. The Na/Al molar ratios for Cu tailings (0.79–1.10) and FA (1.00–1.15) were consistent with those previously reported by the same authors. At early curing stages (1.3 months), G2 exhibited lower Si/Al ratios (1.6–2.9) in comparison to G1 (6.0–8.9), which can be attributed to its higher aluminum content. As curing progressed, both samples displayed fluctuations in the Si/Al ratios, which can be partially attributed to compositional heterogeneity in the analyzed regions, such as the presence of unreacted Cu tailings or FA. By 5.7 months, the Si/Al ratios decreased to 5.1 in G1 and 3.2 in G2, suggesting the formation of more mature and chemically stable gel phases. Likewise, the Na/Al ratios were high at early stages, particularly in G1 (71.0 at 1.3 months), indicating a surplus of unincorporated sodium. The subsequent decline in Na/Al values over time supports the progressive integration of sodium into the aluminosilicate framework, consistent with continued geopolymerization and matrix densification. It was identified that the Si/Al ratio of the Cu tailings was excessively high for the synthesis of activated materials. By incorporating FA, this ratio was reduced, leading to the formation of a denser microstructure [11]. The high silica content present in the tailings leads to an increased SiO_2_/Al_2_O_3_ molar ratio in geopolymers synthesized from these materials, which negatively affects the geopolymerization process [92]. The fluctuations in the values of Si/Al ratios over the different exposure periods could be explained by the fact that the EDS analysis was conducted on localized surfaces, potentially corresponding to either unreacted Cu tailings or FA particles (Table 4).

### 3.3. Mechanical Properties of FA-AAMs

**Figure 7:**
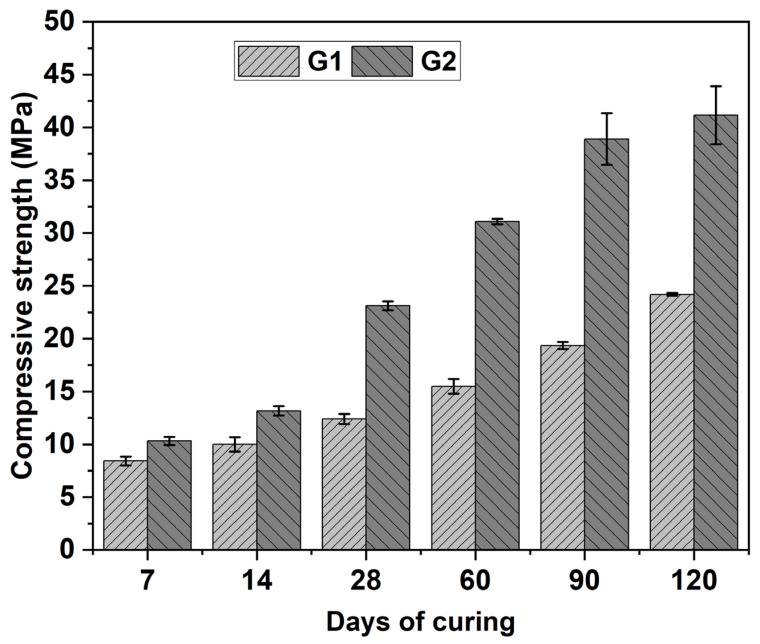
Compressive strength results for FA-AAMs made with Cu tailing (70%) and FA (30%), comparing a G1 and G2 sample over several curing periods.

Figure 7, where bars represent average values and errors bars represent one standard deviation above and below average values (equivalent in Figure 9), illustrates the evolution of compressive strength in G1 and G2 samples over various curing periods. For reference, conventional construction mortars typically exhibit compressive strengths ranging from 5 to 20 MPa, while high-performance mortars designed for structural repair can reach or exceed 30 MPa, depending on the specific application requirements [24]. The compressive strength of the both formulations increased progressively with curing time, with G2 consistently showing higher values than G1. The compressive strength behavior follows a nonlinear trend over curing time, which is typical of blended systems containing both hydraulic and alkali-activated components [26]. In the early stage (up to 28 days), G2 demonstrates a rapid increase in strength, attributed to the synergistic effect of OPC hydration and the initial formation of geopolymeric gels [26,90]. During this period, G1 exhibited a more gradual strength development. The presence of reactive amorphous phases in the precursors appears to play a crucial role in the early strength gain, particularly in the G2 matrix [90]. At 28 days, G1 reached approximately 12.4 MPa, while G2 attained around 23.1 MPa, highlighting the positive impact of OPC incorporation. This enhancement can be attributed to the formation of a denser and more cohesive matrix, promoted by the development of both geopolymeric and cementitious phases [26], as demonstrated by the microstructural analysis presented in Figure 6. Between 28 and 60 days, a transitional phase is observed, during which the continued hydration of OPC in G2 leads to further C–S–H formation and matrix densification, enhancing mechanical performance [7]. In the final stage, from 60 to 120 days, both G1 and G2 continue to exhibit strength development, albeit at a slower rate, reflecting the gradual stabilization and maturation of hydration and geopolymerization products. The G2 formulation reached approximately 41 MPa at 120 days, exceeding the minimum strength thresholds established in standards such as ASTM C90 for masonry and paving units [93]. The G2 formulation can be categorized as a hybrid binder system, as it combines both hydration and geopolymerization mechanisms. This dual-reactivity strategy enhances fresh-state workability and hardened-state performance. Concurrently, the activation of Cu tailings and FA could result in the formation of N–A–S–H gels, which contribute to the progressive development of mechanical strength and long-term durability [26]. Additionally, the calcium content derived from OPC can alter the geopolymerization pathway by favoring the formation of C–A–S–H gels—a phase that structurally bridges the characteristics of both C–S–H and N–A–S–H gels [7]. This assumption is supported by the microstructural changes observed through FESEM analysis. The continued strength development observed beyond 28 days in both G1 and G2 suggests that extended curing times (e.g., up to 120 days) provide a more accurate reflection of the mechanical behavior of alkali-activated systems. This trend is attributed to slower reaction kinetics in low-calcium matrices and, in the case of G2, to the formation of C–S–H and C–A–S–H gels derived from OPC addition. This behavior is consistent with previous studies reporting prolonged strength development in alkali-activated and hybrid binder systems incorporating OPC or other calcium-rich additives [7,11,90]. Based on the results, G2 may be considered suitable for structural or semi-structural uses, while G1 remains a viable alternative for sustainable, low-load applications where extended curing is feasible.

The compressive strength obtained was not intended to meet structural concrete requirements; nonetheless, the results demonstrated sufficient performance for non-structural and moderately loaded applications. These results support the potential use of the material in applications such as partition walls, sub-base layers, insulation panels, and waste containment systems, while simultaneously enabling the reuse of industrial by-products.

**Figure 8:**
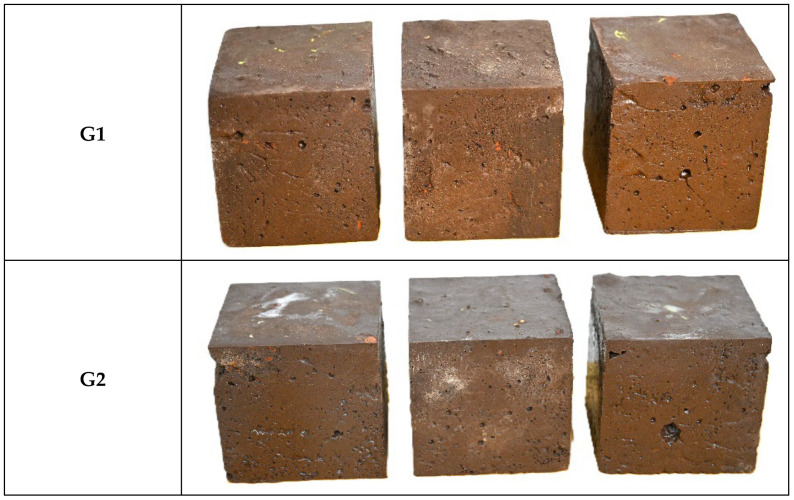
Photographs of triplicate specimens of FA-AAMs with (G2) and without OPC (G1).

Figure 8 presents photographic evidence of the G1 and G2 samples, both of which exhibited visible efflorescence, a phenomenon typically associated with excess alkali or incomplete geopolymerization [94,95]. The composition of the efflorescent material was not determined. The presence of residual alkaline solution could be attributed to the incomplete dissolution of FA during the activation process. Mineralogical analysis indicated that FA contained 1.74% calcite, whereas no calcite was detected in the Cu tailings. Rietveld refinement of XRD data showed 4.98 wt.% calcite in G1 at 1.3 months, while no calcite was detected in G2. The presence of calcite in the G1 sample is likely attributed to the carbonation of lime-containing precursors through reaction with atmospheric CO_2_. It is recognized that calcite formation can increase the volume of hydrates, potentially influencing the material’s dimensional stability [96]. Efflorescence in alkali-activated materials is linked to the migration of unreacted alkalis or soluble salts, which crystallize upon evaporation [94]. However, no structural damage or mechanical deterioration was observed during the 120-day curing period. Strategies to mitigate this issue could include reducing excess alkali content or optimizing curing conditions. Nonetheless, more pronounced surface efflorescence was observed in G2, likely due to calcium availability from OPC and enhanced ion transport through its denser matrix (35% porosity vs. 52% in G1).

Table 3 presents the porosity values of the G1 and G2 samples after 28 days of curing. In conventional systems based on metakaolin or ceramic matrices, a reduction in porosity is typically associated with enhanced mechanical performance [97]. However, this trend does not universally apply to geopolymer systems, particularly those derived from calcined FA, where mechanical strength may increase independently of porosity reductions [94]. In the present study, the G1 sample exhibited a higher porosity (52%) than the G2 sample (35%) at 28 days. This difference is likely attributed to the accelerated hydration kinetics of OPC in G2, which promoted early formation of C–S–H gels, thereby facilitating rapid matrix densification. In contrast, the slower geopolymerization process in G1, which results in the formation of N–A–S–H gels, appears to be less effective in reducing porosity and mitigating shrinkage at early stages. The observed porosity trends are consistent with the mechanical performance of the samples, where the lower porosity of G2 correlates with superior compressive strength. Setting times of G1 and G2 showed that OPC addition in G2 accelerated hydration, which is in line with its role in hybrid AAMs [26]. The limited reactivity of Cu tailings under alkaline conditions was reflected in the performance of both G1 and G2 formulations. Over extended curing periods, both G1 and G2 formulations showed progressive matrix densification and porosity reduction, particularly in G2 due to the presence of OPC (Figure 6). By 5.7 months, G2 exhibited a highly compact and homogeneous structure, correlating with its higher compressive strength (~41 MPa) compared to G1 (~24 MPa). G1 required approximately 24 h to set and exhibited high porosity, while G2 set within 18 h and showed lower porosity, correlating with improved mechanical performance. Although these values are longer than typical setting times observed in conventional cementitious systems, they remain within the acceptable range for certain applications. For cement-based materials, the initial setting time should be no less than 45 min, while the final setting time should not exceed 375 min [98]. The prolonged setting times observed can be attributed to multiple factors, including the chemical composition, mineralogical characteristics, and solubility of the precursor materials, as well as the specific curing conditions employed. Notably, both Cu tailings and FA exhibited LOI values exceeding the 5% threshold established by the applicable Chilean standard for cementitious materials, indicating a considerable presence of organic matter [58]. It is well established that elevated organic content may retard setting and hardening processes due to the generation of porosity through organic matter degradation [9,76]. These LOI values are consistent with reports that organic matter contents above 1% can hinder hydration [76]. Due to poor consistency in pastes made with only Cu tailings, a 70:30 Cu tailings-to-FA ratio was selected. Improvements in strength, phase development, and microstructure confirmed that OPC addition contributed to matrix densification, reduced porosity, and enhanced mechanical performance over time. These findings emphasize the importance of prolonged curing in enhancing microstructural development and mechanical performance in alkali-activated materials.

Extended setting times may be unsuitable for time-sensitive construction applications; however, they can be advantageous in scenarios that allow for prolonged curing, such as waste stabilization, precast element production, or the use of stabilized materials under controlled conditions. In such cases, alkali-activated materials represent a promising and environmentally sustainable alternative to traditional OPC-based systems.

### 3.4. Leaching

**Figure 9:**
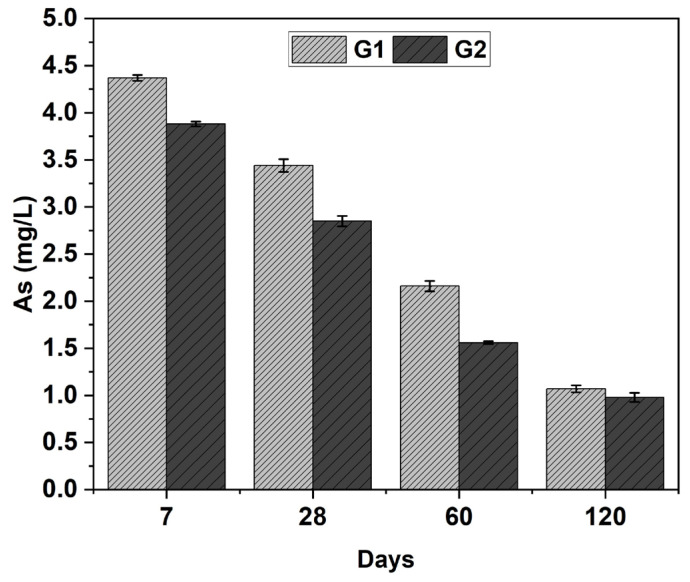
Leached concentrations of As determined from the TCLP test on G1 and G2 samples at various curing times.

The TCLP method [71], although primarily intended for waste classification, was applied here as an indicator of the chemical stability of the developed formulations. While this study did not develop specific tests on chemical resistance or long-term durability, the importance of such assessments is recognized for fully validating the construction potential of alkali-activated materials. Table 5 shows the concentrations of elements leached from the G1 and G2 samples, as well as from the Cu tailings and FA samples determined using the TCLP test. Table 5 also presents the total concentrations of elements contained in the Cu tailings, FA, and OPC samples, as determined by ICP-OES after 120 days. All measured values of PTEs leached after the application of the test were below the maximum allowable concentrations (MAC), confirming compliance with environmental safety standards. Among the analyzed elements, As consistently exhibited the highest concentrations in the leachates. G1 samples released greater amounts of As than G2 samples across all evaluated curing times. In both systems, a progressive reduction in As concentration was observed with increasing curing duration (Figure 9), indicating improved immobilization of this element over time. The Cu tailings used as precursors were also identified as the primary source of As, as they released significantly higher concentrations of As during leaching compared to FA. This inference is corroborated by ICP-OES data (Table 5), which revealed substantially elevated total As content in the Cu tailings—higher than the quantities leached—indicating partial retention within the matrix. These findings support the hypothesis that As leached originates predominantly from the Cu tailings. While As was identified as the primary element released during leaching from the samples, the specific mechanisms responsible for its immobilization—such as incorporation into N–A–S–H or C–A–S–H gels, physical entrapment, or chemical bonding—were not directly analyzed in this study. However, prior research has demonstrated that As retention in alkali-activated materials may occur through various mechanisms, including adsorption onto aluminosilicate gels and precipitation as calcium arsenate phases under alkaline conditions [50].

Geopolymers and hybrid alkali-activated materials are known to stabilize contaminants through mechanisms such as chemical incorporation into aluminosilicate gel networks, precipitation of stable phases, and physical encapsulation. It has been shown that the geopolymer matrix can reduce contaminant leachability due to its dense and cohesive gel structure [50,99]. The potential of this approach as a stabilization technique for mine tailings has been previously validated [99]. In the present study, the incomplete integration of Cu tailings into the geopolymeric framework may have allowed certain mineral phases to remain exposed. This could facilitate the mobilization of arsenate species under alkaline conditions, as As oxyanions are known to exhibit high solubility over a wide pH range. Furthermore, the enhanced leachability of oxyanion-forming elements such as As and V under alkaline activation conditions has been previously reported [74]. However, extended curing periods have been shown to enhance As retention, likely due to increased geopolymer gel formation and microstructural densification that limit contaminant mobility over time [74]. More recent studies have further confirmed the combined environmental and structural performance of geopolymers in effectively immobilizing multiple metal contaminants [8,44]. The observed trends of continuous strength gain, porosity reduction, and matrix densification observed during the curing period suggest favorable durability characteristics. These properties have been strongly linked with enhanced resistance to chemical attack and environmental degradation in alkali-activated systems [92].

For materials intended for construction applications, it is important to assess long-term leaching behavior under conditions that better reflect real-world exposure scenarios. In this context, future studies could develop complementary tests—such as column leaching, diffusion-controlled assays, or dynamic leaching protocols—to more accurately evaluate the long-term release, mobility, and transport of contaminants from alkali-activated matrices.

## 4. Conclusions

This study confirmed the technical and environmental feasibility of using abandoned copper mine tailings (Cu tailings) as precursor in the production of fly-ash-based alkali-activated materials (FA-AAMs). The formulations developed—G1 (without ordinary Portland cement, OPC) and G2 (with 15% OPC)—demonstrated distinct behaviors in terms of setting time, porosity, and mechanical strength. After 120 days, G2 exhibited an average compressive strength of ~41 MPa and G1 reached ~24 MPa. This 71% increase in average strength for G2 was attributed to the synergistic effect of OPC-induced hydration and geopolymer gel formation, supported by field emission scanning electron microscopy (FESEM) and X-ray diffraction (XRD) observations. Additionally, porosity measurements (G1: 52%; G2: 35%) reinforced the correlation between matrix densification and mechanical performance.

Toxicity Characteristic Leaching Procedure (TCLP) leaching tests confirmed that both systems effectively immobilized hazardous elements. Arsenic leaching decreased from 4.4 to 1.01 mg/L in G1 and from 3.8 to 1.0 mg/L in G2 over 5.7-months, remaining well below the regulatory threshold (5.0 mg/L). These results validate the chemical stability of the binders and their potential for use in environmental remediation applications.

However, several limitations were noted. The slow setting time and extended curing requirement of G1 (24 h vs. 18 h in G2) may limit its applicability in time-sensitive construction scenarios. Furthermore, the presence of efflorescence observed in both formulations indicates possible alkali migration, which may negatively impact surface durability over time. These phenomena should be addressed through improved curing protocols or additive selection.

The developed FA-AAMs are most appropriate for non-structural and moderate-load applications, such as sub-base layers, partition walls, or encapsulation blocks. Their ability to immobilize toxic elements while reusing industrial residues positions them as promising materials for waste management and sustainable construction practices, in linear with circular economy principles. Nevertheless, further work should focus on assessing long-term performance, field validation under realistic conditions, and optimization of mix design—particularly in tailoring the Si/Al and Na/Al ratios to maximize durability and minimize environmental risks.

## Figures and Tables

**Table 1:** Formulations used to prepare FA-AAMs.

Mix ID	Cu Tailings (kg/m^3^)	FA (kg/m^3^)	OPC (kg/m^3^)	Water (kg/m^3^)	NaOH (kg/m^3^)	Sodium Silicate (kg/m^3^)	L/S	Si/Al	Si/Na
G1	1260	540	0	3	122	500	0.28	11.4	2.9
G2	1071	459	270	3	122	500	0.28	9.7	3.2

**Table 2:** Summary of chemical, physical, and mineralogical properties of raw materials.

Parameter	Cu Tailing	FA	OPC	Method
SiO_2_ (wt.%)	77.82	55.39	5.83	XRF
Al_2_O_3_ (wt.%)	5.35	13.12	–	XRF
CaO (wt.%)	0.86	9.73	13.87	XRF
Fe_2_O_3_ (wt.%)	3.60	2.88	–	XRF
Na_2_O (wt.%)	0.39	0.49	–	XRF
K_2_O (wt.%)	0.55	0.75	–	XRF
MgO (wt.%)	0.16	0.65	–	XRF
LOI (wt.%)	5.71	11.82	–	XRF
pH	7.5	10.7	–	Direct measurement
Specific gravity	2.49	1.90	–	Pycnometry
SSA (m^2^/g)	0.809	0.534	–	Laser Diffraction
d90 (µm)	105.2	111.2	–	Laser Diffraction
Amorphous content (wt.%)	-	64.4	2.51	XRD (Rietveld)
Main Crystalline Phases	Quartz (64.56 wt.% [00-046-1045] *) Phillipsite-K (7.77 wt.% [00-046-1427] *) Loveringite (5.05 wt.% [00-042-1368] *) Periclase (4.69 wt.% [00-045-0946] *) Lautite (3.39 wt.% [00-039-0393] *)	Quartz (24.37 wt.% [00-046-1045] *) Mullite (4.18 wt.% [00-015-0776] *) Ettringite (2.48 wt.% [00-041-1451] *) Calcite (1.74 wt.% [00-047-1743] *)	Larnite (19.93 wt.% [00-033-0302] *) Titanite (14.73 wt%) Calcite (13.87 wt.% [00-047-1743] *) Gismondine (12.64 wt.% [00-020-0452] *)	XRD (Rietveld)
FESEM Morphology	Irregular, angular particles with coarse texture and voids	Mostly spherical particles, smooth surfaces, some agglomerates	Not characterized in detail	FESEM

Notes: (a) * ICDD—International Centre for Diffraction Data record. (b) LOI: Loss On Ignition. (c) Additional crystalline phases identified in the Cu tailings included: natisite (2.67 wt.% [00-048-1892] *), sturmanite (2.64 wt.% [00-035-0637] *), greigite (2.58 wt.% [00-016-0713] *), grossite (1.73 wt.% [00-046-1475] *), virgilite (1.33 wt.% [00-031-0707] *), birnessite (1.26 wt.% [00-043-1456] *), gypsum (0.87 wt.% [00-033-0311] *), andalusite (0.64 wt.% [00-018-0036] *), colusite (0.43 wt.% [00-044-1474] *), and anatase (0.40 wt.% [00-021-1272] *). (d) Additional crystalline phases identified in the FA included: sylvite (1.65 wt.% [00-041-1476] *), alabandite (0.57 wt.% [00-006-0518] *), thaumasite (0.40 wt.% [00-046-1360] *), cuprite (0.14 wt.% [00-005-0667] *), and briartite (0.05 wt.% [00-026-0527] *). (e) Additional crystalline phases identified in the OPC included: Titanite, syn (14.73 wt.%), spurrite (10.54 wt.% [00-013-0496] *), albite (6.10 wt.% [00-041-1480] *), quartz (5.83 wt.% [00-046-1045] *), anorthite (2.92 wt.% [00-041-1481] *), tenorite (2.16 wt.% [00-048-1548] *), manganocummingtonite (1.71 wt.% [00-023-0603] *), manganocolumbite, Fe-rich (0.75 wt.% [00-045-1360] *), muscovite-3T (0.19 wt.% [00-007-0042] *), and cassiterite, syn (0.09 wt.% [00-041-1445] *).

**Table 3:** Summary of structural, physical, and mechanical properties of G1 and G2 formulations.

Parameter	G1	G2
Amorphous content (1.3 months) (wt.%)	33.25	41.81
Porosity (28 days, MIP) (%)	52.0	35.1
Setting time	24 h	18 h
Compressive strength (28 days → 120 days) (MPa)	12.4 → 24.2	23.1 → 41.2
Main Crystalline Phases (XRD) after 5.7 months **	Quartz (65.5 wt.% [00-046-1045] *) Muscovite (8.8 wt.% [00-007-0025] *) Clinoclase (6.54 wt.% [00-037-0447] *) Albite (5.1 wt.% [00-041-1480] *) Thenardite (4.6 wt.% [00-037-1465] *)	Quartz (63.2 wt.% [00-046-1045] *) Albite (11.7 wt.% [00-041-1480] *) Clinoclase (5.49 wt.% [00-037-0447]) Thenardite (5.3 wt.% [00-037-1465] *) Muscovite (5.0 wt.% [00-007-0025] *)
FESEM Morphology at 5.7 months	Compact matrix with visible voids, partially reacted tailings.	Highly compact, homogeneous structure, minimal voids.

Notes: (a) * ICDD—International Centre for Diffraction Data record. (b) ** The values for the other curing times are presented in Tables S1 and S2 can be found in the additional files. (c) Additional crystalline phases identified in the G1 sample: rodolicoite (3.08 wt.% [00-029-0715] *), tetrahedrite (1.99 wt.% [00-042-0561] *), rectorite (1.83 wt.% [00-029-1495] *), potassium aluminum sulfate hydroxide (1.02 wt.% [00-047-1884] *), anatase (0.88 wt.% [00-021-1272] *), aluminum fluoride (0.30 wt.% [00-044-0231] *), natroalunite (0.17 wt.% [00-041-1467] *), birnessite (0.11 wt.% [00-043-1456] *), and chalcopyrite (0.10 wt.% [00-037-0471] *). (d) Additional crystalline phases identified in the G2 sample: rectorite (2.34 wt.% [00-029-1495] *), chalcopyrite (2.58 wt.% [00-037-0471] *), rodolicoite (2.00 wt.% [00-029-0715] *), birnessite (0.78 wt.% [00-043-1456] *), aluminum fluoride (0.71 wt.% [00-044-0231] *), natroalunite (0.63 wt.% [00-041-1467] *), and anatase (0.39 wt.% [00-021-1272] *).

**Table 4:** EDS chemical composition of G1 and G2 samples at different curing times.

Months	Type	Figure	Point	Weight %/Error %								
				Na	Al	Si	S	K	Ca	Fe	Si/Al	Na/Al
1.3	G1	A	1	32.7/7.08	0.5/24.26	2.8/6.97	1.2/9.62		0.2/24.70	0.2/35.50	6.0	71.0
			2	19.4/8.11	1.5/10.43	13.6/5.19	9.9/5.41	0.5/21.54	1.0/13.54	1.7/12.81	8.9	12.7
	G2	B	1	1.0/14.87	0.6/10.05	28.8/3.23	-	-	0.2/24.53	-	47.3	1.6
			2	17.0/7.34	4.2/6.46	12.2/4.90	3.0/5.65	0.1/59.47	1.6/4.88	0.2/26.47	2.9	4.0
4.8	G1	C	1	18.3/6.62	2.3/11.58	35.8/4.29	-	-	3.3/20.88	-	15.4	7.9
			2	2.4/13.24	0.8/16.76	24.7/4.09	2.2/14.55	0.7/43.32	-	1.8/18.54	31.7	3.1
			3	7.7/7.32	3.7/7.29	26.7/4.10	-	-	-	-	7.3	2.1
	G2	D	1	21.5/5.71	0.4/30.82	1.7/9.2	-	-	1.7/14.60	-	4.2	53.7
			2	10.1/6.07	0.9/9.67	6.6/4.53	-	-	3.8/9.09	-	7.7	11.7
5.3	G1	E	1		1.3/9.42	31.6/3.53	-	-	-	-	24.3	0.0
			2	11.7/6.61	2.6/8.64	26.9/4.03	-	-	1.3/26.98	-	10.5	4.6
			3	8.6/6.84	6.4/5.63	29.6/3.94	-	1.2/19.76	1.9/18.52	-	4.6	1.3
	G2	F	1	24.1/5.78	3.6/7.44	9.0/4.97	4.3/8.31	-	4.2/10.50	-	2.5	6.7
			2	7.8/7.31	1.1/12.00	11.9/4.54	-	-	15.3/5.47	-	11.2	7.3
5.7	G1	G	1	12.2/6.66	2.4/9.49	27.0/4.07	-	-	3.5/14.23	-	11.2	5.1
	G2	H	1	8.9/7.25	2.8/8.00	14.7/4.43	2.0/14.19	0.8/28.76	19.4/5.39	-	5.3	3.2

**Table 5:** Leached concentrations (TCLP) from G1, G2, Cu tailings, and FA, and total elemental contents (ICP-OES) of raw materials.

	Leached Concentrations Obtained from the TCLP Test (mg/L)				Determinations by ICP-OES in Raw Material Samples			
Total Element Concentration	G1 (120 d)	G2 (120 d)	Cu Tailing	FA	Cu Tailing (mg/kg)	FA (mg/kg)	OPC (mg/kg)	MAC (mg/L)
As	1.1	1.0	4.054	<0.002	3098	<2.00	2.66	5.0
Cr	<0.007	<0.007	0.0235	0.0593	13.8	8.13	35.50	5.0
Hg	<0.002	<0.002	<0.002	<0.002	n.d *	n.d *	n.d *	0.2
Pb	<0.051	<0.051	<0.051	<0.051	51.10	<30.00	<30.00	5.0
Se	0.072	0.074	0.065	0.072	2.42	16.00	6.62	1.0
Ba	<1.009	<1.009	<1.009	<1.009	4301	297.7	101.80	100.0
Cd	<0.045	<0.045	<0.045	<0.045	1.14	<0.50	4.57	1.0
Ag	<0.032	<0.032	<0.032	<0.032	4.70	<2.00	2.66	5.0

Notes: The total concentration (mg/kg) of the other elements contained in the raw materials, determined by ICP-OES, was as follows: (a) Cu tailings: Fe (19,236), Cu (379.0), Ca (4056), Ti (42.4), Al (1567), Mg (509.1), Na (1471), K (472.8), Zn (28.7), Sr (78.8), Mn (124.8), P (93.3), Sb (98.0), V (10.4), Sn (19.3), Mo (<4.00), Li (<2.00), Be (<0.50), Co (<2.00), Tl (<50.0), Bi (<20.0). (b) FA: Ca (71,902), Al (8044), Fe (8383), K (1339), Na (1013), P (302.5), Mn (129.2), Ti (412.3), Mg (2222), Cu (25.8), Zn (37.7), V (48.6), Sb (45.4), Sr (177.5), Li (15.8), Mo (12.7), Ni (13.9), Sn (17.9), Be (3.35), Co (<2.00), Bi (<20.0), Tl (<50.0). (c) OPC: Ca (313,274, Al (16,968), Fe (16,417), Na (5633), K (5251), P (1749), Mg (6141), Ti (1283), Sr (269.1), Mn (344.8), Zn (58.0), Cu (90.3), Ni (44.3), V (106.4), Sn (39.2), Li (24.1), Co (9.11), Sb (10.1), Mo (<4.00), Bi (<20.0), and Tl (<50.0). (d): The total concentrations of leachates determined by the TCLP method at various curing times are presented as follows: At 7 days: G1—As: 4.4 mg/L, Cr: <0.007 mg/L, Hg: <0.002 mg/L, Pb: <0.051 mg/L, Se: 0.203 mg/L, Ba: <1.009 mg/L, Cd: <0.045 mg/L, Ag: <0.032 mg/L; G2—As: 3.88 mg/L, Cr: <0.007 mg/L, Hg: <0.002 mg/L, Pb: <0.051 mg/L, Se: 0.203 mg/L, Ba: <1.009 mg/L, Cd: <0.045 mg/L, Ag: <0.032 mg/L; At 28 days: G1—As: 3.4 mg/L, Cr: <0.007 mg/L, Hg: <0.002 mg/L, Pb: <0.051 mg/L, Se: 0.006 mg/L, Ba: <1.009 mg/L, Cd: <0.045 mg/L, Ag: <0.032 mg/L; G2—As: 2.9 mg/L, Cr: <0.007 mg/L, Hg: <0.002 mg/L, Pb: <0.051 mg/L, Se: 0.009 mg/L, Ba: <1.009 mg/L, Cd: <0.045 mg/L, Ag: <0.032 mg/L; At 60 days: G1—As: 2.2 mg/L, Cr: <0.007 mg/L, Hg: <0.002 mg/L, Pb: <0.051 mg/L, Se: 0.066 mg/L, Ba: <1.009 mg/L, Cd: <0.045 mg/L, Ag: <0.032 mg/L; G2—As: 1.6 mg/L, Cr: <0.007 mg/L, Hg: <0.002 mg/L, Pb: <0.051 mg/L, Se: 0.061 mg/L, Ba: <1.009 mg/L, Cd: <0.045 mg/L, Ag: <0.032 mg/L; (e) * n.d: not detected.

## Data Availability

The original contributions presented in this study are included in the article/Supplementary Material. Further inquiries can be directed to the corresponding author.

## Supplementary Materials

The following supporting information can be downloaded at: https://www.mdpi.com/article/10.3390/ma18173926/s1, Table S1: Mineralogical composition of G1 sample after several curing time. Table S2: Mineralogical composition of G2 sample, after several curing time.

## Abbreviations

The following abbreviations are used in this manuscript:

AAMs	Alkali-activated materials
C–(A–)S–H	Calcium (alumino) silicate hydrate
C-(N)-A-S-H	Calcium (alkali) aluminosilicate hydrate
CaO	Calcium oxide
C–S–H	Calcium silicate hydrate
EDS	Energy Dispersive Spectroscopy
FA	Fly ash
FA-AAMs	Fly-ash-based alkali-activated materials
FESEM	Field Emission Scanning Electron Microscopy
HAAC	Hybrid alkali-activated cement
LOI	Loss on Ignition
MgO	Magnesium oxide
N-A-S-H	Sodium aluminosilicate hydrate
OPC	Ordinary Portland cement
PDS	Particle size distribution
SCM	Supplementary cementitious materials
SH	Sodium hydroxide
SS	Sodium silicate
SSA	Specific surface area
TCLP	Toxicity Characteristic Leaching Procedure
XRF	X-ray diffraction
