# Supplementary material for: Use of Abandoned Copper Tailings as a Precursor to the Synthesis of Fly-Ash-Based Alkali Activated Materials
Source: Materials (Basel). 2025 Aug 22;18(17):3926. doi: 10.3390/ma18173926 (PMC12429135; doi:10.3390/ma18173926)
Supplement: Supplementary file 1 [file materials-18-03926-s001.zip › materials-3587985-supplementary.pdf]

Annex

Table S1. Mineralogical composition of G1 sample after several curing time.

Compound	Formula	Curing time (months)			
		1.3	4.8	5.3	5.7
Albite, Ca-rich, ordered [00-041-1480]	$(Na,Ca)Al(Si,Al)_3O_8$	6.55	8.11	13.26	5.11
Anhydrite, syn [00-037-1496]	$CaSO_4$	1.22			
Annite-1M [00-042-1413]	$KFe^{2+}_3(AlSi_3O_{10})(OH)_2$	3.16			
Birnessite, syn [00-043-1456]	$Na_{0.55}Mn_2O_4\cdot1.5H_2O$	2.81	0.68	0.33	0.11
Calcite [01-088-1808]	$CaCO_3$	4.98			
Greigite [00-016-0713]	Fe_3S_4	2.28		1.69	
Pinakiolite [00-036-0413]	$(Mg,Mn)_2Mn(BO_3)_2$	1.37			
Quartz, syn [00-046-1045]	SiO_2	38.55	71.70	41.85	65.53
Rectorite [00-029-1495]	$K_{1.2}Al_4Si_8O_{20}(OH)_4\cdot4H_2O$	1.83	1.23	2.74	1.83
Thenardite, syn [00-037-1465]	Na_2SO_4	1.79		4.83	4.57
Walthierite [00-047-1833]	$BaAl_6(SO_4)_4(OH)_{12}$	2.08			
Ferberite [00-046-1446]	$FeWO_4$	0.12			
Clinoclase [00-037-0447]	$Cu_3(AsO_4)(OH)_3$		3.51	9.91	6.54
Aluminum Fluoride [00-044-0231]	AlF_3		0.59	0.75	0.30
Anatase, syn [00-021-1272]	TiO_2		0.28	0.57	0.88
Barite, syn [00-024-1035]	$BaSO_4$		5.00		
Muscovite-1M, syn [00-007-0025]	$KAl_2(AlSi_3O_{10})(OH)_2$		2.76	11.21	8.78
Potassium Aluminum Sulfate Hydroxide [00-047-1884]	$KAl_3(SO_4)_2(OH)_6$		0.41	2.45	1.02
Rodolicoite, syn [00-029-0715]	$FePO_4$		2.47	2.06	3.08
Tetrahedrite, syn [00-042-0561]	$Cu_{12}Sb_4S_{13}$		2.90	1.77	1.99
Mawsonite [00-030-0486]	$Cu_6Fe_2SnS_8$		0.35		
Chalcopyrite [00-037-0471]	$CuFeS_2$				0.10
Natroalunite [00-041-1467]	$NaAl_3(SO_4)_2(OH)_6$				0.17
Grossular [00-039-0368]	$Ca_3Al_2(SiO_4)_3$			1.20	
Vaterite [00-033-0268]	$CaCO_3$			5.14	
Famatinite, syn [00-035-0581]	Cu_3SbS_4			0.25	
Amorphous material		33.25			

Table S2. Mineralogical composition of G2 sample, after several curing time.

Compound	Formula	Curing time (months)			
		1.3	4.8	5.3	5.7
Albite, Ca-rich, ordered [00-041-1480 (I)]	$(Na,Ca)Al(Si,Al)_3O_8$	4.64	12.12	15.53	11.65
Anhydrite, syn [00-037-1496]	$CaSO_4$	0.19		1.26	
Annite-1M [00-042-1413 (I)]	$KFe^{2+}{}_3(AlSi_3O_{10})(OH)_2$	1.45			
Birnessite, syn [00-043-1456]	$Na_{0.55}Mn_2O_4{\cdot}1.5H_2O$	2.07	0.13	0.42	0.78
Greigite [00-016-0713 (I)]	Fe_3S_4	0.28			
Pinakiolite [00-036-0413 (I)]	$(Mg,Mn)_2Mn(BO_3)_2$	3.32			
Quartz, syn [00-046-1045]	SiO_2	36.14	55.12	38,54	63.17
Rectorite [00-029-1495]	$K_{1.2}Al_4Si_8O_{20}(OH)_4{\cdot}4H_2O$	2.28	0.84	2.23	2.34
Thenardite, syn [00-037-1465]	Na_2SO_4	1.50		3.77	5.27
Walthierite [00-047-1833 (I)]	$BaAl_6(SO_4)_4(OH)_{12}$	3.58			
Carlinite, syn [00-029-1344 (I)]	Tl_2S	0.35			
Clinoclase [00-037-0447]	$Cu_3(AsO_4)(OH)_3$	1.27	5.23	6.48	5.49
Troilite-2H [00-037-0477]	FeS	1.11		0.22	
Alabandite, syn [00-006-0518]	MnS		1.54	0.08	
Aluminum Fluoride [00-044-0231]	AlF_3		1.37	0.30	0.71
Anatase, syn [00-021-1272]	TiO_2		0.40	0.82	0.39
Barite, syn [00-024-1035]	$BaSO_4$		5.81		
Braunite-2Q [00-041-1368]	$Mn^{2+}Mn^{3+}{}_6(SiO_4)O_8$		1.74	1.21	
Magnesite, syn [00-008-0479 (I)]	$MgCO_3$		4.21		
Muscovite-1M, syn [00-007-0025 (I)]	$KAl_2(AlSi_3O_{10})(OH)_2$		5.56	8.01	5.00
Periclase, syn [00-045-0946]	MgO		0.55		
Potassium Aluminum Sulfate Hydroxide [00-047-1884 (I)]	$KAl_3(SO_4)_2(OH)_6$		1.22	3.06	

Rodolicoite, syn [00-029-0715]	$FePO_4$		1.07	2.54	2.00
Tetrahedrite, syn [00-042-0561 (C)]	$Cu_{12}Sb_4S_{13}$		2.06	2.16	
Huntite [00-014-0409 (I)]	$Mg_3Ca(CO_3)_4$		1.05		
Dolomite [00-036-0426]	$CaMg(CO_3)_2$			3.81	
Spherocobaltite, syn [00-011-0692 (I)]	$CoCO_3$			3.29	
Hematite, syn [00-033-0664]	Fe_2O_3			1.02	
Chalcopyrite [00-037-0471]	$CuFeS_2$			5.24	2.58
Natroalunite [00-041-1467 (I)]	$NaAl_3(SO_4)_2(OH)_6$				0.63
Amorphous material		41.81			
